# Nano–bio interaction between human immunoglobulin G and nontoxic, near-infrared emitting water-borne silicon quantum dot micelles†

**DOI:** 10.1039/d3ra00552f

**Published:** 2023-02-20

**Authors:** Shanmugavel Chinnathambi, Naoto Shirahata, Mahima Kumar, Subramani Karthikeyan, Katsuhiko Abe, Vaijayanthi Thangavel, Ganesh N. Pandian

**Affiliations:** a Institute for Integrated Cell-Material Sciences (WPI-iCeMS), Institute for Advanced Study, Kyoto University Kyoto 606-8501 Japan CHINNATHAMBI.Shanmugavel.8s@kyoto-u.ac.jp NAMASIVAYAM.ganeshpandian.5z@kyoto-u.ac.jp; b International Center for Young Scientists, National Institute for Materials Science (NIMS) 1-2-1 Sengen Tsukuba 305-0047 Ibaraki Japan; c Graduate School of Chemical Sciences and Engineering, Hokkaido University Sapporo 060-0814 Japan; d International Center for Materials Nanoarchitectonics (WPI-MANA), NIMS Namiki Tsukuba 305-0044 Japan SHIRAHATA.Naoto@nims.go.jp; e Department of Physics, Chuo University 1-13-27 Kasuga, Bunkyo Tokyo 112-8551 Japan; f Centre for Healthcare Advancement, Innovation and Research, Vellore Institute of Technology Chennai 600 127 India

## Abstract

In recent years, the field of nanomaterials has exponentially expanded with versatile biological applications. However, one of the roadblocks to their clinical translation is the critical knowledge gap about how the nanomaterials interact with the biological microenvironment (nano–bio interactions). When nanomaterials are used as drug carriers or contrast agents for biological imaging, the nano–bio interaction-mediated protein conformational changes and misfolding could lead to disease-related molecular alterations and/or cell death. Here, we studied the conformation changes of human immunoglobulin G (IgG) upon interaction with silicon quantum dots functionalized with 1-decene, Pluronic-F127 (SiQD-De/F127 micelles) using UV-visible, fluorescence steady state and excited state kinetics, circular dichroism, and molecular modeling. Decene monolayer terminated SiQDs are accumulated inside the Pluronic F127 shells to form SiQD-De/F127 micelles and were shown to bind strongly with IgG. In addition, biological evaluation studies in cell lines (HeLa, Fibroblast) and medaka fish (eggs and larvae) showed enhanced uptake and minimal cytotoxicity. Our results substantiate that engineered QDs obviating the protein conformational changes could have adept bioefficacy.

## Abstract 2

We prepared Pluronic-F127 coated silicon quantum dot micelles and examined their interaction with Human IgG. Furthermore, the micelles were used to image cells as well as medaka (*Oryzias latipes*) eggs and larvae without causing cytotoxicity.

## Graphical abstract

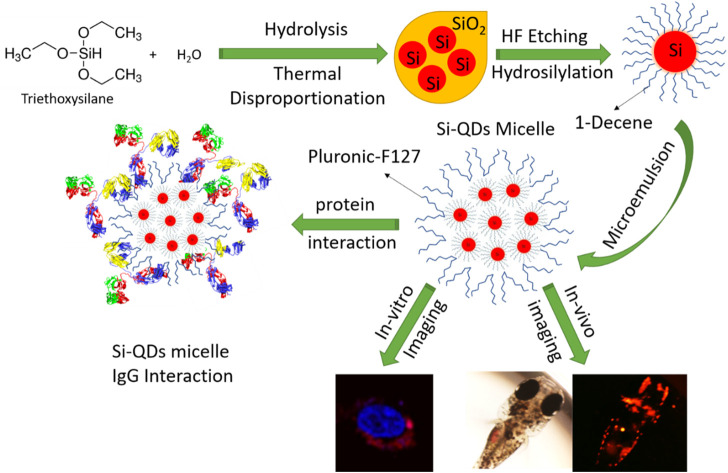

## Introduction

Recent advances in precision medicine primarily rely on developing nanomaterials as diagnostics and therapeutics. In particular, several functionalized nanoparticles have been developed as contrasting agents for *in vivo* and *in vitro* imaging. In recent decades, biologists have been interested in near-infrared (NIR) fluorescent imaging because NIR light can penetrate human tissues (>700 nm) without inducing pathogenic molecular alterations.^1–3^ Therefore, NIR-based fluorescent probes are preferred to visualize the morphology of cells and even organelles.^4^ Semiconductor quantum dots (QDs) play a significant role in biomedical applications such as real-time tissue imaging agents, biosensors and therapeutic agents.^5,6^ Compared to commercial fluorescent probes, QD probes exhibit better stability against photobleaching, high quantum yield, tunable emission, and broad absorption spectral ranges.^7,8^ However, it is difficult to dissolve semiconductor QDs in water; hence, it is necessary need to functionalize the surface of the QD probes with water-soluble materials.^9^ While extensive studies have been focused on the imaging efficacy of functionalized QD probes, only a few studies have been conducted on their interaction with biomacromolecules.^10,11^ Nanomaterial interaction with the biological microenvironment (nano–bio interaction) is critical to harness the excellent fluorescent properties of QDs and use them in deep-tissue imaging, drug delivery, and intra-cellular organelle tracking.^12,13^

**Fig. 1:**
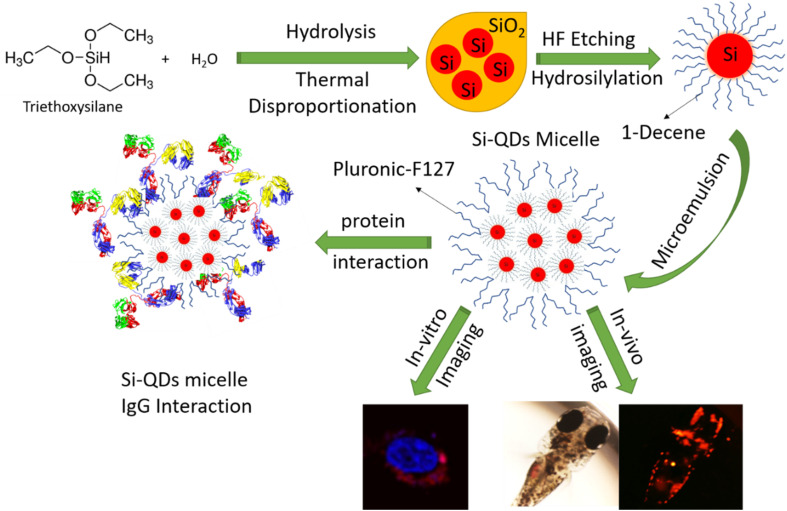
The schematic diagram shows the silicon quantum dots production from the triethoxysilane precursor, hydrosilylation with 1-decene and functionalize with Pluronic-F127. The prepared SiQD-De/F127 micelle used as a biocompatible staining agent in an *in vitro* and *in vivo* model. Also, a two-dimensional picture shows IgG interaction on the surface of the SiQD-De/F127 micelle.

The multi-modality imaging capability of QDs has been extensively harnessed as theranostic agents in chronic systemic inflammatory diseases such as ‘Rheumatoid Arthritis (RA)’. Patients with RA are known to have elevated levels of immunoglobulins than the control population. In particular, immunoglobulin G (IgG), the second most abundant plasma protein in human blood, is known to be 0.6 g L^−1^ higher in positive RA than the controls.^14^ Human IgG has a molecular weight of about 150 kDa and comprises two heavy and light chains linked by disulfide bridges.^15^ Nanoparticles (NP) interact with IgG after injection into the bloodstream and often comprise protein corona.^16,17^ In nonspecific binding, IgG undergoes conformational changes with different hydrophobic and hydrophilic surfaces due to the tendency for the nanoparticle micelles to find a more favorable energy state.^18^ When NPs like QD probes are injected into the bloodstream, the heavy metal ions are released and induce toxicity.^19^ After intravenous injection, biomolecules, primarily plasma proteins, cover the surfaces of NPs to form a protein corona.^20,21^ Concurrently, the adsorption of the protein on the corona micelles alters their secondary or tertiary structures, leading to toxicity.^22^ Cukalevski *et al.* studied protein-induced NP aggregation and discovered that IgG drives NP aggregation in blood serum and induces it in an unexpectedly concentration-dependent manner.^23^ Later, Hassan *et al.* investigated interactions between 13 immunoglobulin isotypes from human, bovine, and murine blood and gold nanoparticles.^24^ Lin *et al.* demonstrated that IgG effectively enhances the PL intensity and stability of amphiphilic poly(acrylic acid) -coated CdSe/ZnS core–shell QDs.^25^ Silicon QDs (Si-QDs) gets proclaimed to have better clinical translation prospects in RA as bioimaging agents owing to their biocompatible properties. However, until now, no studies have been available to show the interaction of Si-QDs or their functionalized derivatives with biomolecules like IgG. Here, we prepared non-toxic (heavy metal ions free), water-borne NIR Si-QDs to elucidate the interaction mechanism with human IgG (Fig. 1). Furthermore, we verified their cellular uptake and viability with the live cells (HeLa and human dermal fibroblasts) and medaka fish (egg and larvae) as a proof-of-concept study to verify their biocompatibility as imaging agents in the *in vitro* and *in vivo* models.

## Experimental section

### Materials

1-Decene, IgG from human serum was purchased from Sigma-Aldrich (I4506-10MG). Triethoxysilane (TES) received from Tokyo Chemical Industry. Wako Pure Chemical Industries (Tokyo, Japan) provided all other reagents and chemicals. Hydrofluoric acid was purchased from Kanto Chemical Japan, which is < 100 ppm metal impurity and 46–51% aqueous solution. We used deionized water from Sartorius (arium 611 UV) purifier (Sartorius AG, Goettingen, Germany). Normal human fibroblast cell lines (JCRB0527) and HeLa (JCRB9004) were purchased from the JCRB cell bank.

### Production of Si-QDs

16 mL of TES was placed in a two-neck flask placed in an ice bath and stirred under an Ar atmosphere. Hydrochloric solution of pH 3 (32 mL) was added dropwise to the TES solution while stirring vigorously under Ar flow. The use of acidic water was needed to prevent deprotonation of the TES during the hydrolysis. We filtered the solution under reduced pressure. A white powder (hydrogen silsesquioxane) was rinsed with Milli-Q water until pH 7 and then dried overnight in a vacuum. After being moved to a quartz crucible, the dried powder was heated in a vacuum furnace. Five gas purges were then performed using 5%-H_2_/95%-Ar gas. The powder was thermally disproportionated at 1150 °C for 3 hours under a 5%-H_2_/95%-Ar atmosphere. The resulting brown powder was SiQD dispersed in a SiOx matrix.

### Decene-terminated SiQD production

The SiQD/SiOx powder, 300 mg, was pulverized in an agate mortar with a pestle. The fine powder was placed in a 50 mL Teflon container. By agitating for 80 minutes in an acidic solution containing 8 mL of ethanol and 16 mL of 48% HF solution, the SiQDs were freed from the oxide matrix. The hydrogen-terminated SiQDs were collected at 15 000 rpm centrifugation for 30 minutes and washed with ethanol twice and acetonitrile in that order. The resultant product dispersed in 1-decene was transferred to a two-neck flask under Ar atmosphere. The final product was heated at 170 °C for 2 hours in an atmosphere of Ar after being purged with Ar for 15 minutes at room temperature. Immediately following the reaction, the mixture was cooled to ambient temperature. High-performance liquid chromatography (HPLC) was used to purify the resulting decene-terminated SiQDs (SiQDs-De). Following drying under vacuum, the purified QDs were dissolved in toluene.

### Production of SiQD-De/F127 micelles

10 mL of acidic water (0.1 N HCl) was used to dissolve 200 mg of pluronic F127, then stirred for one hour. In a vial, 1 mg of SiQDs-De was mixed with Pluronic F127 solution containing a screw bottle. The bottle was shaken for a few minutes vigorously to achieve emulsion formation; then, the screw bottle was opened to the air until it evaporated the toluene layer completely (∼48 hours) (Fig. S1(a)†). The leftover sample was treated with sonication for a short period and then transferred to a 14 KDa dialysis tube. Two hours later, the unreacted Pluronic F127 and HCl were removed by dialysis against water. The resultant sample looks like a milky solution. Further dilution with water is necessary for biological applications.

### Characterization

JASCO V-650 spectrometer was used for UV-visible absorption spectra. Rigaku Smart Lab X-ray diffractometer (XRD) was used to obtain the SiQDs-De diffraction pattern. Tecnai G2 F30, a high-resolution transmission electron microscope (HR-TEM) with 300 kV, was used to capture the crystalline lattice structure of SiQDs-De. Before observations, the Pluronic-coated SiQDs-De samples were deposited onto the ultrathin copper grid. The excitation and emission spectra of the SiQDs-De and Pluronic coated SiQDs-De were measured by InGaAs detector for NIR on a NanoLog spectrofluorometer (Hamamatsu Photonics, Japan). The power lamp source is a 450 W xenon arc lamp. IgG was excited with a 279 nm LED source, and the Pluronic coated SiQDs-De were excited with a 370 nm pulsed spectral LED. The experimental decay curve fitting values were decided based on the *χ*^2^ value, which is near to value 1. Using an integrating sphere C9920-03G system from Hamamatsu Photonics in Japan with a xenon lamp light source (150 W), the photoluminescence quantum yield (PLQY) was measured. The spectropolarimeter (model J-725; Jasco, Tokyo, Japan) measured circular dichroism spectra using a 1 mm quartz cuvette and a 100 nm min^−1^ scan rate at ambient temperature. We measured each spectrum three times and acquired an average to finalize the results. Each CD data was measured three times and averaged. BESTSEL web server was used for protein secondary structure predictions.

### Particle size analysis by AF4

The Asymmetric-flow field flow fractionation (AF4) apparatus is attached to an HPLC manual injection valve (Wyatt Technology) and includes a 20 μL sample loop made of stainless steel; this machinery consists of an isocratic pump (1260 series (G1310B), Agilent Technologies, Santa Clara, CA). A channel for AF_4_ separation (Eclipse, Wyatt Technology, Santa Barbara, CA) using 5 kDa molecular weight cut-off filter with regenerated cellulose membrane, MALS detector from Dawn 8+, Wyatt Technology, A diode array detector in the UV-vis range (1260 DAD (G1315D), Agilent Technologies). Before AF4 analysis, we used a 0.22 μm membrane filter and sonicated and 10 mM PBS was used as an eluent. The investigation was carried out using an injection flow rate of 0.2 mL min^−1^ and 0.25 mL min^−1^. About 50 μL of the sample was injected into the system for micelles analysis. Usually, after sample injection, focus and axial flow were generally in opposition to one another to condense the micelles sample into a tiny space. We need to wait about 10 minutes, utilizing a cross-flow of 0.25 mL min^−1^, to reach the equilibrium stage. Around 0.5 mL min^−1^ of detector flow rate was continued. ASTRA software has been used for all AF4 analyses (version 5.3.4.15, Wyatt Technology).

### Cell culture and cytotoxicity assay

For *in vitro* studies, we used (HeLa and human dermal fibroblasts) that were grown in a 75 cm^2^ flask at 37 °C in humidified air containing 5% CO_2_ and supplied with 10% fetal bovine serum, 5000 U per mL penicillin, and 50 μg mL^−1^ streptomycin. Both cell lines are maintained with the DMEM medium. We got all our tissue culture supplies from Fisher Scientific. The cells were grown onto a 96-well plate at a density of 5000 cells per well for the cell cytotoxicity assessment. After a 24 hours preincubation period, SiQD-De/F127 micelles were applied to cells and the cell viability was measured after incubating them at 24 and 48 h using the Cell Counting Kit-8 (Dojindo Laboratories, Osaka, Japan). With a conventional microplate reader, absorbance at 450 nm was determined (MTP-880Lab; Corona, Hitachinaka, Japan). Experiments on cell viability were carried out in triplicate, and the outcomes were displayed as ± mean standard deviation. Compared to untreated control cells, the percentage of cell cytotoxicity was displayed.

### Live-cell imaging

To visualize the SiQD-De/F127 micelles in the live cells, we used a confocal laser scanning fluorescence microscope (FluoView, FV10i, Olympus) with UV laser excitation. We seeded HeLa and Fibroblast cells in a 35 mm dish, and 24 hours later, 100 μg mL^−1^ nanomaterials were added to a glass bottom dish as a final concentration. After a 24 hours incubation period, the cells were washed in PBS and fixed for 10 minutes in 4% paraformaldehyde before confocal imaging. The cell nucleus was stained with Hoechst 33 342 dye. Integrated fluorescence intensities from each cell were calculated using Image-J software.

### Medaka fish culture

For *in vivo* studies, we used medaka fish (*Oryzias latipes*) embryos and larvae. The mature *Oryzias latipes* were purchased from a local fish farm (Higo-pet, Kyoto Prefecture) and were subjected to artificial reproductive conditions in a freshwater glass aquarium maintained at 25 °C in a 10 hours dark and 14 hours light cycle. As described before, the spawned eggs were collected, maintained and monitored in a Petri-dish.^26^ The eggs were seeded in triplicates onto a 96-well plate (one per well) and the treatment was done on a 4 days-old egg and was monitored every day until day 28. The larvae hatched out of the egg around day 17 were maintained and monitored in a separate glass tank. Image-J software calculated mean fluorescence intensities from medaka fish embryos and larvae.

### Molecular docking studies

ChemDraw was used to draw the F-127 compound's initial chemical structure. The steepest descent approach and conjugate gradient approach were used to reduce the energy of the chemical structure 5000 times in total, an impact panel integrated into the Schrodinger software. The protein data bank portal was used to download the IgG (PDB ID: 6KA7). The two protein structures that were obtained were optimized and reduced using the force field from the OPLS 2005 software of the Protein Preparation Wizard panel. The steps for preparing the protein file were as follows: initially, remove all water from around the protein molecule; (ii) add hydrogen atoms to the appropriate protein structure; (iii) assign coulomb charges; and (iv) native (co-crystal) molecules were taken out of the corresponding protein molecules; and (v) minimized the energy of the structure. After the docking experiments using the induced fit panel have generated a maximum of 10 possible postures, the best docking position is chosen based on glide energy and docking score.^27–32^

## Results and discussion

### Synthesis and characterization of SiQD-De/F127 micelle

**Fig. 2:**
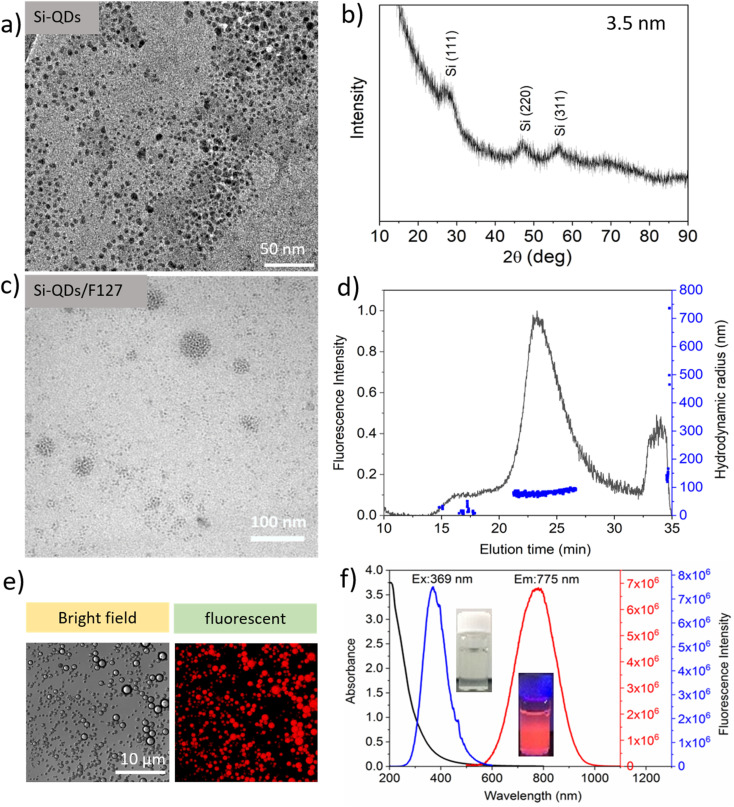
TEM images of SiQD-De (a), XRD spectrum of SiQD-De (b), TEM images of SiQD-De/F127 micelles (c), AF_4_ analysis shows a micelle size of between 30 to 80 nm (d), Fluorescent bright field and fluorescent images of the microscale micelle (e), Fluorescent absorption, excitation and emission spectra of micelles (f).

As per our prior procedure, we produced a water-borne micelle with a Pluronic F127 shell and an assembled SiQD core that is terminated by decene monolayers.^5^ SiQD termination with alkyl monolayers significantly improved PLQYs.^33–35^ By wrapping the SiQD-De inside a Pluronic F127 molecule, the SiQD-De was transported from toluene to water. Pluronic F127 were chosen as coating material in this study for several reasons. First, Pluronic block copolymers are amphiphilic synthetic polymers composed of hydrophilic poly(ethylene oxide) (PEO) blocks and hydrophobic poly(propylene oxide) (PPO) blocks arranged in a triblock configuration: PEO–PPO–PEO. The hydrophobic PPO segments include a hydrophobic core that serves as a microenvironment for lipophilic drug. The hydrophilic PEO corona inhibits aggregation, protein adsorption, and reticuloendothelial recognition (RES).^36^ The resulting SiQDs-De/F127 was then purified using dialysis and a 14 kDa MWCO tube. According to TEM images (Fig. 2a), SiQD-De micelles are well distributed. The average size (3.5 nm) of SiQD-De was calculated by X-ray diffraction (Fig. 2b). After surface modification with Pluronic-F127, the sizes of materials increased between 30 to 80 nm (Fig. 2c and S1(b)†). In addition, with TEM images, AF_4_ analysis also suggests that most of the micelles present nearly 80 nm hydrodynamic radius (Fig. 2d). The TEM and AF4 results are also supported by dynamic light scattering analysis (85 nm) (Fig. S2†). To confirm the fluorescence nature of the materials, we prepared SiQD-De/F127 micelles and investigated with confocal fluorescence microscopy. The red color fluorescence intensity was produced by micelles at the microscale, as shown in Fig. 2e. The SiQD-De/F127 micelles emit the light at 775 nm when excited with 369 nm. In addition, we could observe the wide-ranging absorption spectrum and narrow excitation spectrum of the micelles (Fig. 2f). SiQDs emission ranges were controlled by QDs size. In addition, with 3.5 nm (775 nm emission) SiQDs, we synthesized 4.8 and 4.0 nm SiQDs with fluorescence emissions of 886 and 800 nm (Fig. S3†). Finally, small-size SiQDs are used to prepare micelles, which are then used for cell imaging and IgG interaction studies.

### Interaction between Si-QD-De/F127 and IgG: UV-vis absorption study

**Fig. 3:**
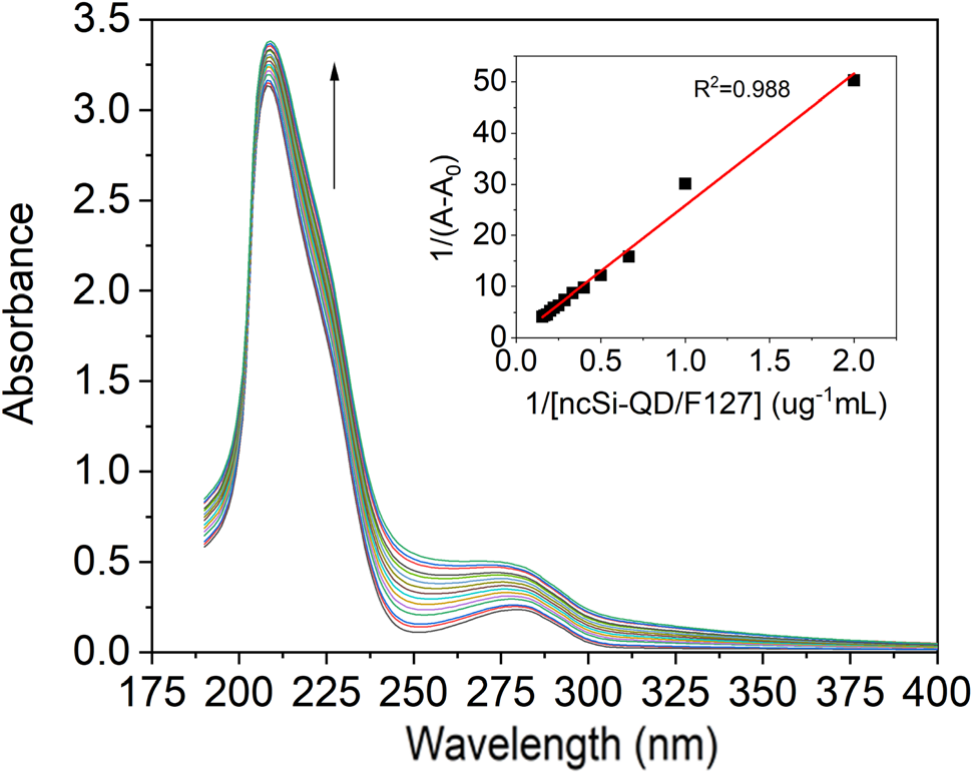
IgG (1 μM) absorption spectra with increasing concentration (0.154, 0.167, 0.182, 0.200, 0.222, 0.250, 0.286, 0.333, 0.400, 0.500, 0.667, 1.000, 2.000 μg mL^−1^) of SiQD-De/F127 micelles. Benesi–Hildebrand graph for IgG as a factor of micelles concentration is displayed in the insets.

UV-Vis absorption spectroscopy is an easy and ideal tool to investigate the interaction between proteins and small molecules or nanomaterials. When exposed to an external source, amino acid residues are highly sensitive to the local environment. For spectroscopy investigations, aromatic amino acids are more sensitive, especially tryptophan. Such microenvironment changes the absorption maxima and spectral shift of the IgG. Due to Si-QD-De/F127, the absorption spectra were measured between 190 and 400 nm to examine the peptide strands of IgG. We recorded IgG (1 μM) absorption spectra (Fig. 3), in the presence of increasing concentrations of SiQD-De/F127 (0–2.0 μg mL^−1^). Around 220 and 278 nm, two prominent bands developed; the first is caused by the π–π* transition of polypeptide backbone structure C=O transition, while the second is due to aromatic amino acids, particularly for tryptophan of IgG. After adding SiQD-De/F127, the polypeptide backbone region shows minimal absorption changes, while the aromatic amino acid region shows considerable spectral changes. Which means microenvironment of aromatic amino acids are more sensitive than peptide backbone while interact with foreign molecule or nanomaterials.^37^

The above results confirm that ground-state complex formation is possible between IgG and SiQD-De/F127 micelles.

Using eqn (1), the degree of complex formation between IgG and SiQD-De/F127 was assessed.

1 IgG + SiQD-De/F127 ⇌ [IgG]: SiQD-De/F127

where *K* is the provided value for the association constant

2 *K* = [IgG: SiQD-De/F127]/[IgG] [SiQD-De/F127]

Using the Benesi–Hildebrand relation, the binding constants of complex formation were calculated from the alteration in the absorption peak's intensity at 278 nm.^38^

3 1/(*A*_obs_ − *A*_0_) = 1/(*A*_c_ − *A*_0_) +1/*K*_app_ [*A*_c_ − *A*_0_] 1/[ SiQD-De/F127]

where *A*_0_ and *A*_obs_ are the IgG solution's absorption at 1 μM and IgG varying in the concentration of SiQD-De/F127, separately. *A*_c_ represents the final absorption of the ligated IgG. The binding constant (*K*_app_) for the IgG–SiQD-De/F127 complex is reported to be 3.11 × 10^4^ M^−1^, and the plot of 1/(*A*_obs_ − *A*_0_) against 1/[SiQD-De/F127] is shown to be linear.

### A steady-state fluorescence analysis of the interaction between SiQD-De/F127 and IgG

**Fig. 4:**
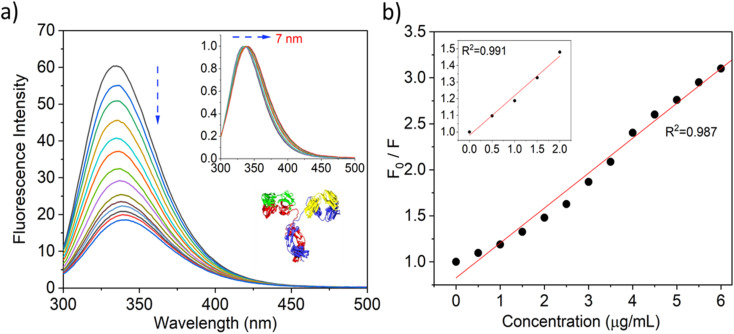
Quenching of IgG tryptophan fluorescence while interacting with increasing concentrations (0, 0.5, 1.0, 1.5, 2.0, 2.5, 3.0, 3.5, 4.0, 4.5, 5.0, 5.5, 6.0, 6.5 μg mL^−1^) of SiQDs/F127 micelles (a). Fluorescence quenching spectra and red spectral shift (inset) (b). Stern–Volmer graph of IgG with and without SiQDs/F127 micelles. Insets show low concentration levels of SiQDs/F127 micelles. Protein data bank information is used to create the IgG crystal structure, PDB ID: 6KA7 (4a, inset).

To evaluate the fluorescence quenching nature of the IgG while interacting with SiQD-De/F127, we used fluorescence steady and excited state spectroscopy. Usually, tryptophan gives intrinsic fluorescence of more than 95% compared with other aromatic amino acids like tyrosine and phenylalanine. The fluorescence spectra of IgG were observed with an incremental concentration of SiQD-De/F127 (Fig. 4a). With rising SiQD-De/F127 concentrations, the fluorescence intensity of IgG significantly reduced, and a redshift (∼7 nm) was observed, which indicated that SiQD-De/F127 interacted with IgG, causing the fluorescence chromophore of IgG to be positioned in a more hydrophobic environment.

The following Stern–Volmer equation helps to demonstrate the quenching mechanism.^39,40^

4 *F*_0_/*F* = 1 + *K*_q_*τ*_0_[Q] = 1 + *K*_sv_[Q]

*F*_0_ denotes the fluorescence intensity in the absence of a quencher. With the quencher, *F* represents the fluorescence intensity. *K*_q_ indicates quenching constants of IgG. The average lifetime (*τ*_0_) of IgG indicates 2 × 10^8^ s. The Stern–Volmer quenching constant is denoted by *K*_sv_, while the quencher concentration is represented by [Q]. Fig. 4b specifies the fluorescence quenching of IgG by SiQD-De/F127. The reason behind the fluorescence quenching is the complex formation between IgG and SiQDs-De/F127. The *K*_q_ value, which is higher than the biopolymer's diffusion rate constant (1.47 × 10^13^ L^−1^ mol^−1^ s^−1^), further supported the static quenching theory. In addition, with the above results, time-resolved fluorescence analysis was performed to verify the types of quenching mechanisms.

The following equation can be used to determine the binding site (*n* = 0.98) and binding constant (*K* = 2.89 104 Lmol^−1^) based on Fig. 4b.^41^

5 log[(*F*_0_ − *F*)/*F*] = log *K*_a_ + *n* log[Q]

Table 1 summarizes the calculated parameters. When we used a lower concentration of nanomaterials (<2.0 μg mL^−1^), a linear relationship was obtained (see Fig. 4, inset) between IgG and SiQD-De/F127, which causes ground state complex formation or static quenching. PL quenching in high concentration leads to dynamic or collisional quenching.

**Table 1:** Binding values of IgG during interaction with SiQD-De/F127 micelles^a^

	*K*_sv_ (μg^−1^ mL)	*K*_q_ (μg^−1^ mL s^−1^)	*τ*_0_ (ns)	*n*	*R* ^2^
IgG (constant)/micelles	0.29 ± 0.06	1.05 × 10^10^	2.80	0.98	0.99

a *K*_SV_ represents the Stern–Volmer constant, *K*_q_ indicates the biomolecular quenching rate constant, *τ*_0_ denotes lifetime, and *n* represents the number of the binding site in the IgG. *R*^2^ shows the goodness of fit.

**Fig. 5:**
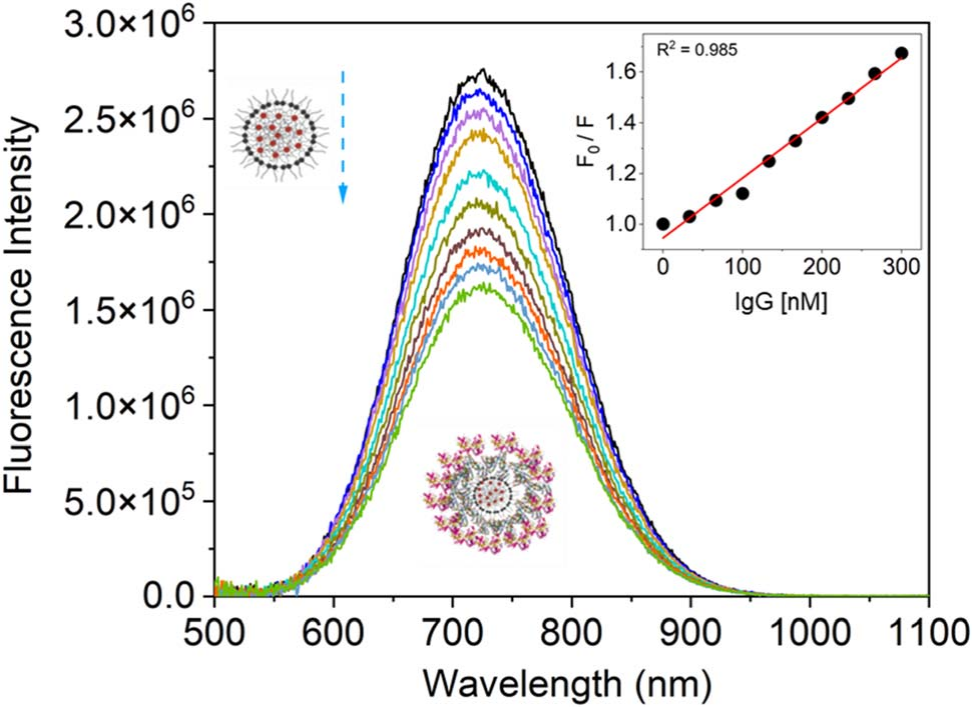
Fluorescence spectra of SiQDs-De/F127 micelles after adding IgG in incremental amounts (0, 33.33, 66.66, 99.99, 133.32, 166.65, 199.98, 233.31, 266.64, 300.00 nM). SiQDs/F127 micelles fluorescence emission spectra and their accompanying Stern–Volmer graphs are displayed in both the absence (block line) and presence of IgG.

With the addition of IgG gradually (0–300 nM), we later observed the fluorescence quenching behavior of the SiQD-De/F127 micelles. The results are shown in Fig. 5, and the Stern–Volmer plot shown inset. The goodness of fit value (*R*^2^ > 0.98) shows linear quenching trends when more IgG is added, indicating that the PL intensity of SiQD-De/F127 micelles is degraded. The calculated parameters are tabulated in Table 2. The *K*_q_ value exceeds the top limit of the scatter collision quenching constant (2.0 × 10^10^ M^−1^ s^−1^), showing that the static quenching process dominates the SiQD-De/F127 fluorescence quenching.^42^

**Table 2:** Binding values of SiQD-De/F127 micelles during interaction with IgG^a^

	*K*_sv_ (L^−1^ M^−1^)	*K*_q_ (L^−1^ M^−1^)	*τ*_0 (_μs)	*n*	*R* ^2^
Micelles (constant)/IgG	0.179 × 10^7^	2.29 × 10^10^	78.16	0.881	0.985

a *K*_SV_ represents the Stern–Volmer constant, *K*_q_ indicates the biomolecular quenching rate constant, *τ*_0_ denotes lifetime, and *n* represents the number of the binding site in the IgG. *R*^2^ shows the goodness of fit.

### A time-resolved fluorescence analysis of the interaction between IgG and SiQD-De/F127

**Fig. 6:**
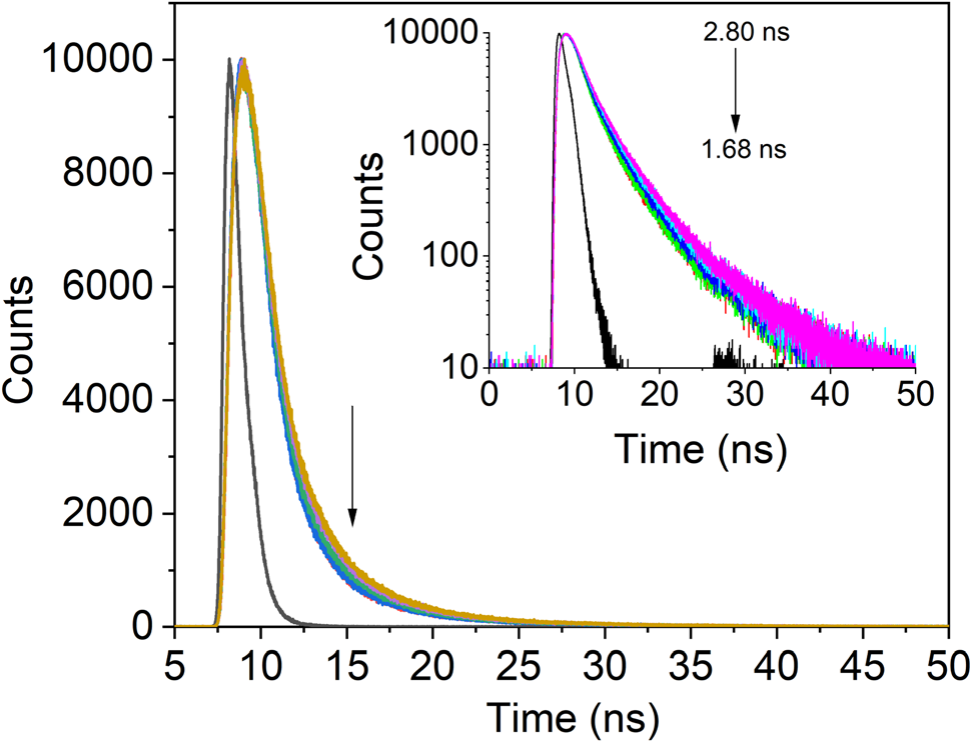
IgG's fluorescence lifetime profile with the addition of SiQDs/F127 micelles (0, 3.33, 6.66, 10.00, 13.33 μg mL^−1^). Arrows show that lifetime decreases as micelles are gradually added.

The PL decay curves for IgG with increasing concentrations of SiQD-De/F127 were measured to clarify the PL dynamics of the IgG- SiQD-De/F127 complex. Fig. 6 demonstrates the fluorescence decay profile of IgG before and after SiQD-De/F127 addition. IgG exhibits single exponential decay in dilute solutions and in the presence of SiQD-De/F127. There are significant variations in the lifetime of IgG (from 2.8 ns to 1.68 ns) as SiQD-De/F127 concentration is raised. This result suggests that fluorescence quenching happened by static mechanism and supports the ground state complex formation. The estimated decay time is summarized in Table 3.

**Table 3:** The addition of SiQD-De/F127 micelles gradually alters the tryptophanyl fluorescence lifetime of IgG

	Micelles conc. (μg mL^−1^)	*α*_1_ × 10^3^	*α*_2_	*α*_3_	*τ*_1_ (ns)	*τ*_2_ (ns)	*τ*_3_ (ns)	*τ*_0_ (ns)	*χ* ^2^
IgG	0 (Only protein)	6.55	3.23 × 10^−3^	6.55 × 10^−4^	8.36	2.73	5.79	2.79	1.06
	03.33	7.54	3.31 × 10^−3^	1.17 × 10^−3^	6.64	2.10	4.09	2.23	1.13
	06.66	7.59	2.35 × 10^−3^	2.17 × 10^−3^	6.36	1.55	2.94	1.84	1.11
	10.00	7.93	2.88 × 10^−3^	1.99 × 10^−3^	6.05	1.43	2.88	1.72	1.10
	13.33	5.82	2.39 × 10^−3^	4.12 × 10^−3^	5.70	2.66	1.15	1.68	1.09

Fig. 5 and 6 fitted with a triexponential function as shown by eqn (6).^43^6
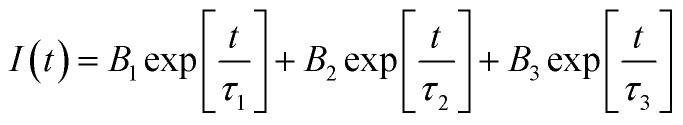


In the equation above, *B*_1_, *B*_2_, and *B*_3_ stand in for the amplitudes of each component, while PL lifetimes for the first, second, and third components, respectively, are represented by 1, 2, and 3. When SiQD-De/F127 was gradually added, the radiative lifetime of IgG was reduced. A triexponential function was used to fit the IgG decay curve, yielding an estimated average decay time of 2.79 ns and time constants of 8.36, 2.73, and 5.79 ns. The average lifetime dropped to 1.68 ns under the influence of 13.33 g mL^−1^ micelles. The minor difference in PL decay time indicates that the lower concentrations of the SiQD-De/F127 addition do not affect the IgG PL decay dynamics.

**Fig. 7:**
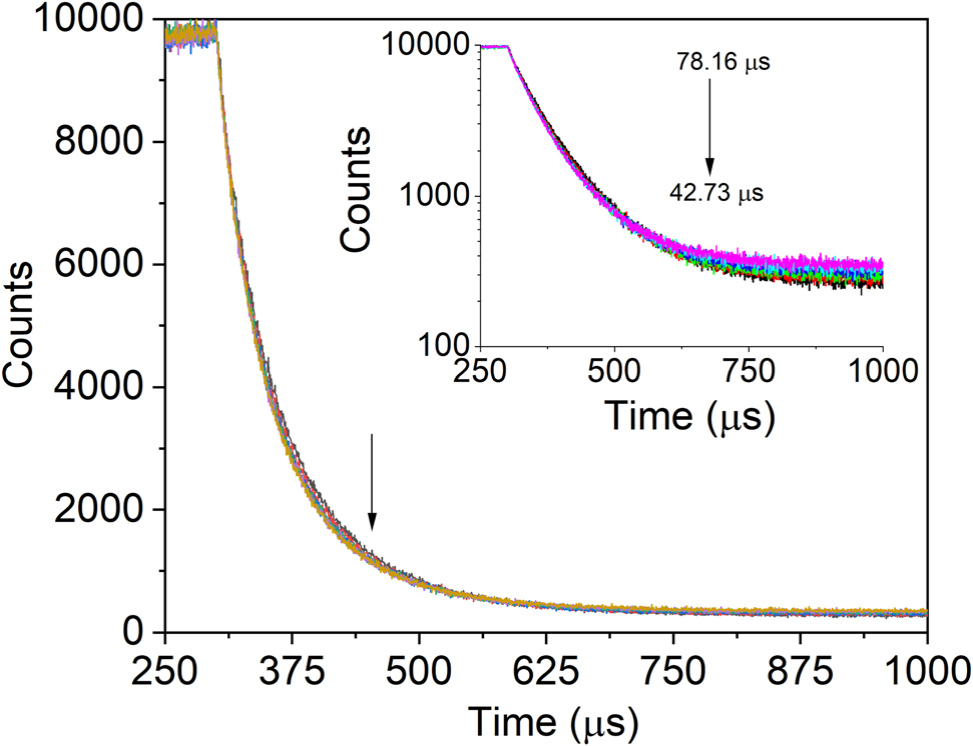
SiQDs/F127 micelles fluorescence lifetime profile with the addition of IgG (0, 33.33, 66.66, 99.99, 133.32, 166.65 nM). After interacting with IgG, SiQDs/F127 micelles fluorescence lifetime is displayed in the insets.

A biexponential function was used to match the measured PL decay patterns for the SiQD-De/F127 with the progressive addition of IgG (Fig. 7). The fitting parameters are listed in Table 4. As the concentration rose, IgG's degradation time revealed decreasing tendencies (arrows, Fig. 7, inset). A bi-exponential fit with lifetime components of 26.1 and 80.3 s was used to estimate the average decay time, which was found to be 78.1 s. The average lifetime of the micelles dropped to 42.7 s after the serial addition of IgG. The tryptophanyl fluorescence's PL dynamics differed from the IgG incremental addition's effect on the micelles PL decay.

**Table 4:** SiQD-De/F127 micelles PL lifetime is altered by adding IgG in nanomolar concentrations

	Protein conc. (nM)	*α*_1_ × 10^3^	*α*_2_ × 10^3^	*τ*_1_ (μs)	*τ*_2_ (μs)	*τ*_0_ (μs)	*χ* ^2^
IgG	0 (only micelle)	1.07	8.44	26.1	80.3	78.1	0.97
	33.33	2.32	7.03	24.5	71.3	66.5	0.99
	66.66	2.48	6.58	25.6	71.1	65.6	1.02
	99.99	2.53	6.56	24.8	68.7	63.3	1.04
	133.32	2.12	6.91	22.7	65.1	61.0	0.92
	166.65	2.05	7.29	18.3	45.5	42.7	0.99

When we modify the surface of the SiQD-De with Pluronic F127, the PLQY reduction is very fast. Adsorption of the IgG on the SiQD-De/F127 surface changed the molecular structure of Pluronic F127 micelles. Using two parameters, *k*_r_ for radiative processes and *k*_nr_ for nonradiative processes, PLQY (*η*) is defined as *η* = *k*_r_/(*k*_r_ + *k*_nr_), where *k*_r_ = 1/*τ*_r_ and *k*_nr_ = 1/*τ*_nr_. This process may diminish the micelle protecting performance against oxygen and water, leading to mild oxidation of QDs. Because the oxidized QD surface acts as a nonradiative channel and reduces the PL decay time, this procedure raises the value of *τ*_nr_.^44^

### IgG secondary structure analysis

One of the sensitive techniques to track the structural changes of the protein is circular dichroism (CD) spectroscopy. We captured the SiQD-De/F127- and SiQD-De/F127-IgG CD spectra. The secondary structure of IgG was calculated using an online software called BeStSel (http://bestsel.elte.hu/index.php) created for secondary structure identification and folding recognition from protein CD spectra.^45,46^

**Fig. 8:**
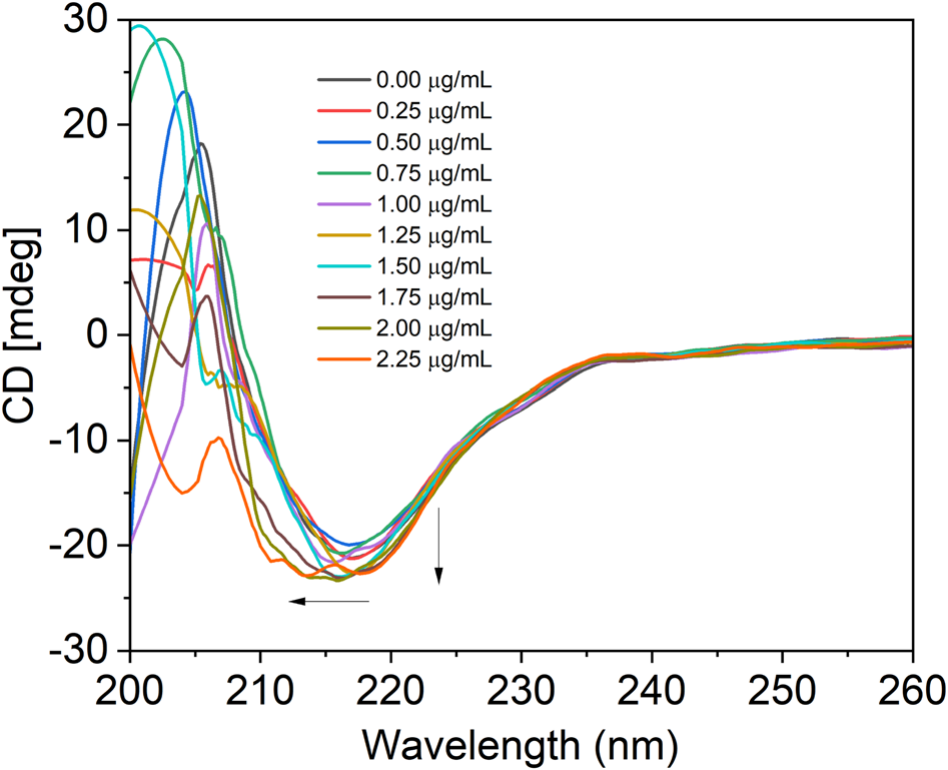
CD spectra reveal the secondary structure and conformational stability of the IgG following their association with SiQDs/F127 micelles (0, 0.25, 0.50, 0.75, 1.00, 1.25, 1.50, 1.75, 2.00, 2.25 μg mL^−1^). Arrows describe the type of wavelength shift or change that occurs when micelles are combined with IgG.

With the gradual addition of SiQDs/F127 micelles, we evaluated IgG CD spectra from 200 to 260 nm. The IgG and its combination with SiQDs/F127 micelles CD spectra are shown in Fig. 8. A negative peak is present between 210 to 225 nm due to the β-sheet secondary structure of IgG.^47^ The spectral shape and wavelength shift happened when adding 1.25 μg mL^−1^ of micelles, indicating IgG denaturation. Native IgG (0.1 M) includes 100% parallel beta sheets; however, as micelles (0–2.25 μg mL^−1^) were added, the value steadily dropped to 7.96%. Table 5 shows the percentages of parallel and anti-parallel beta-sheet changes and other secondary structure changes, such as a loop. Up to 1.5 μg mL^−1^ of micelles, in addition, the secondary structure of IgG does not alter when we go more than that; we observe irregular/loop structure in the Table 5. This result supports the utility of SiQDs/F127 micelles in biomedical applications as they could still have excellent biocompatibility even when utilized in a lower concentration. In addition, shorter *in vivo* circulation times further substantiate their biocompatibility.

**Table 5:** Analysis of IgG's secondary structure upon interactions with SiQD-De/F127 micelles

IgG (0.1 μM) + micelle (μg mL^−1^)	Anti (%)	Parallel (%)	Others (%) irregular/loop
0.00	0	100	0
0.25	0	100	0
0.50	22.29	77.71	0
0.75	25.96	74.04	0
1.00	29.41	70.59	0
1.25	32.16	67.84	0
1.50	48.65	51.35	0
1.75	42.78	24.06	33.17
2.00	53.35	12.74	33.91
2.25	18.27	7.96	73.77

### Cell viability and cellular uptake

**Fig. 9:**
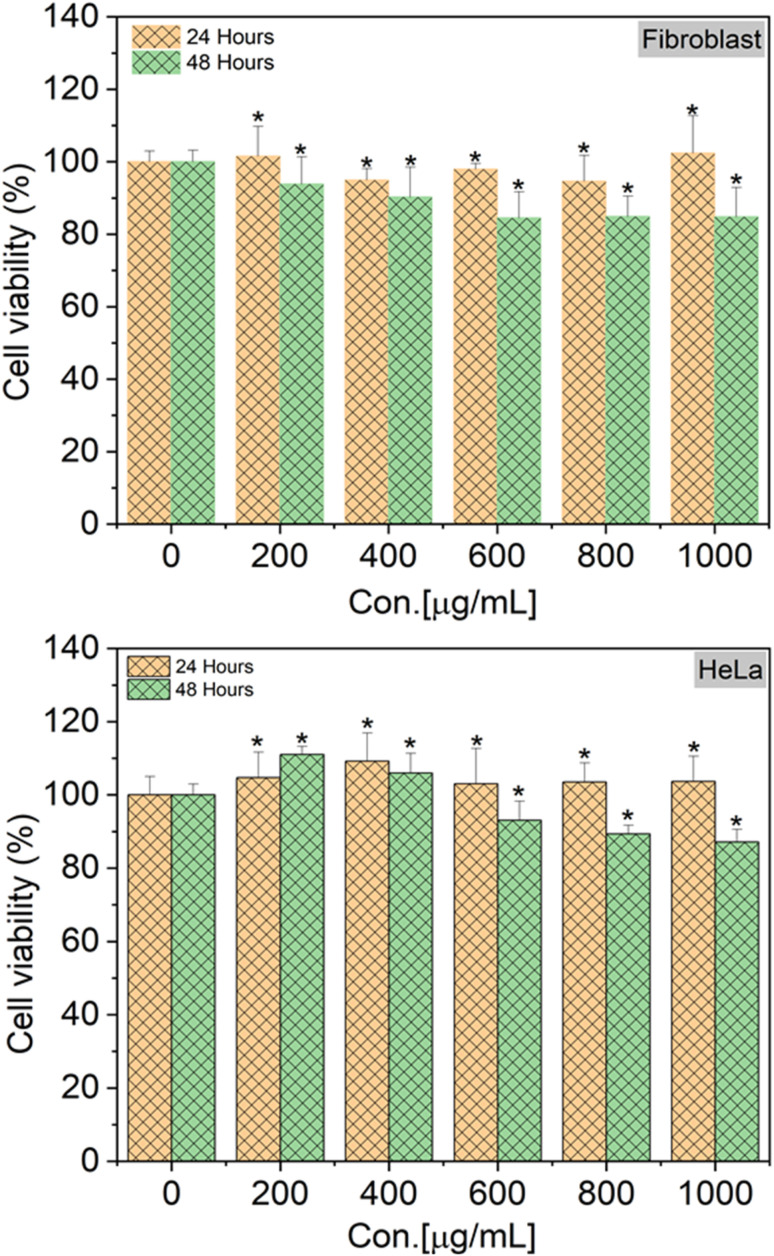
Cell viability assay using SiQDs/F127 micelles with human dermal fibroblasts (fibroblast) and HeLa cells. Cell viability was represented as mean ± S.E. (*n* = 3). Treated groups showed statistically significant differences from the control group by the unpaired two-tailed Student's *t*-test (*p < 0.05).

To verify the biocompatibility of SiQDs-De/F127 micelles in the *in vitro* models, we performed cellular viability and *in vitro* imaging studies using HeLa cells and human dermal fibroblasts (HDFs) as representative cell lines. As shown in Fig. 9, HeLa cells and HDFs were almost 100% viable after 24 h incubation, as no cytotoxicity was observed even with 1 mg mL^−1^ of SiQDs-De/F127 micelles. Interestingly, even with 48 h incubation, only a limited amount of toxicity (10%) was observed in both cell lines. Together, the cell-viability studies substantiate that the functionalized SiQDs-De/F127 micelles were relatively less hazardous than the heavy-metal QDs^19^ and are expected to have excellent biocompatibility for diagnostic studies using live cells. Pramanik *et al.* evaluated toxicity assay of SiQDs and CdSe and CdSe/ZnS using bacteria models *Shewanella oneidensis* and *Bacillus subtilis.*^48^ The effects of SiQDs on two different bacteria, one Gram-negative and the other Gram-positive, were studied and compared to the effects of two traditional Cd-based QDs (CdSe and CdSe/ZnS). In colony counting assays, the SiQDs had no effect on the viability of bacteria cells, whereas the CdSe QDs had significant dose-dependent toxic effects on the bacteria. The above findings support the SiQDs-De/F127 micelles cell viability assay performed on HeLa and Fibroblast cells.

**Fig. 10:**
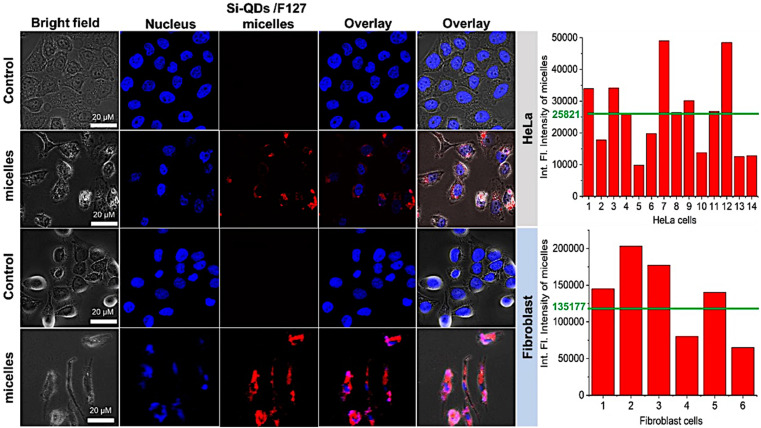
*In vitro* imaging studies using SiQDs/F127 micelles with human dermal fibroblast (Fibroblast) and HeLa cells. The bar charts show the integrated fluorescence intensity of individual cells and the green line represents the average intensity caused by the micelles. Triplicate wells were used for each cell line.

For *in vitro* imaging studies, we employed a fluorescence confocal microscope to visualize the uptake in the HeLa and fibroblast cell culture media to validate that SiQDs/F127 micelles are nontoxic (∼85% cell viability) even after cellular localization at 1 mg mL^−1^ concentration. As shown in Fig. 10, the SiQDs/F127 micelles did not enter the cell nucleus but were dispersed throughout the cytoplasm. So far, it has been widely accepted that the cellular uptake of nanoparticles is influenced by a number of factors, including nanoparticle size, concentration, and surface charge.^49–51^ Phatvej *et al.* demonstrated an endocytosis pathway for alkyl-capped silicon quantum dots using various cell lines, including HeLa.^52^ According to the references, SiQDs/F127 micelles are taken up by HeLa and Fibroblast cells *via* endocytosis. After endocytosis, the micelle will be localized in the early endosome and later translocation to late endosome.^53^ Finally, SiQDs/F127 micelles spread throughout the cytoplasm. From the fluorescence intensity of the cells, the endocytosis faster in Fibroblast than HeLa.

### SiQDs-De/F127 uptake in an *in vivo* model

**Fig. 11:**
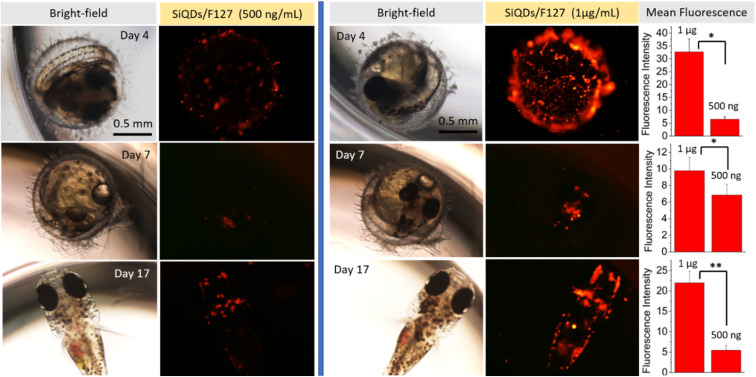
SiQDs/F127 micelle uptake by Medaka fish (egg and larvae) with two different concentrations of 500 ng mL^−1^ and 1 μg mL^−1^. The fluorescence intensity plot shows a difference between two micelle concentrations. Three eggs were used for each micelle concentration ± S.E. (*n* = 3). Asterisks indicates *p < 0.05 or **p < 0.01.

We chose to verify the *in vivo* staining capacity of SiQDs-De/F127 micelles by evaluating their uptake in the Japanese medaka fish model (embryo and larvae). After collecting the eggs from the fish tank, we transferred them to 96-well plates and incubated them with 200 μL of sterile, nuclease-free water. We then added 500 ng and 1 μg mL^−1^ of SiQDs-De/F127 micelles and performed confocal microscopy studies after 24 hours of incubation to examine if micelles could be uptaken by medaka eggs. As seen in Fig. 11, SiQDs-De/F127 micelles are effectively localized inside the medaka fish egg and larvae without any vectors or transfection agents. A significant (*P* < 0.05) difference in the fluorescence intensity could be observed with low (500 ng mL^−1^) and high (1 μg mL^−1^) concentrations of SiQDs-De/F127 micelles on days 4, 7, and 17 (Fig. 11). Even at higher concentrations (1 μg mL^−1^), the SiQDs-De/F127 micelles-treated fish embryo developed into normal larvae and exhibited excellent fluorescence distribution thereby substantiating their biocompatibility in the *in vivo* imaging. Furthermore, Kwok *et al.* evaluated silver nanoparticle uptake by medaka larvae and concluded most of the particles interact with the skin surface and are taken up *via* the gills.^54^ Using polystyrene microspheres, Kashiwada *et al.* evaluated four types of nano-sized distribution in medaka eggs/larvae, and most of the particles enter through adsorption or accumulation.^55^ Based on the references, the possibility of the SiQDs-De/F127 micelles uptake by medaka eggs/larvae is adsorption/bioaccumulation or entering *via* the gills. Silver nanoparticle contains Ag, which is toxic to the living organism,^56^ but we demonstrated SiQDs-De/F127 micelles is non-toxic to the cells and living organism.

### Molecular docking analysis

Molecular docking is an excellent resource for comprehending intricate biological systems at the atomic level. Following the experimental evidence supporting F127's binding in the IgG complex system, we performed molecular docking studies to gain deeper insights into how the F127 complex interacts with the microenvironment of IgG. After identifying the active site residues using blind docking, the F127 and IgG complex systems were shown to have additional induced fit docking on the active site residues from that complex. The induced fit docking simulation produced 10 distinct binding poses; for further studies of the F127 complex IgG system, the gliding energy and docking score were used to determine the best positions (Table S1†).

**Fig. 12:**
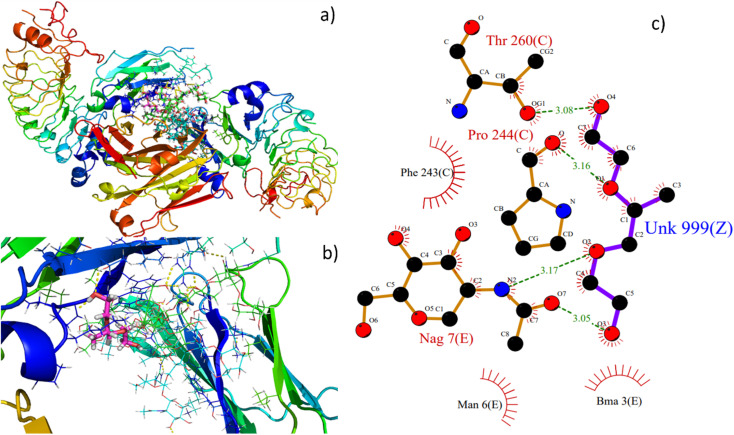
Best binding poses of the pymol view of (a) F127- IgG complex, (b) F127 binding site, (c) hydrogen bond interactions of ligand with the residues represented in green colored lines and nearby hydrophobic residues in red colored lines. In ligplot, ligand is represented in sticks.

As seen in Fig. 12a and b, the optimal site for the F127 complex, are shown to bind with the IgG system. The binding energy and docking score of the F127 complex with the IgG system are −26.7 kcal mol^−1^ and −2.9 kcal mol^−1^, respectively, and three hydrogen bonding formations were found between F127-IgG complex. As seen in Fig. 12c, a OH and O atom in F127 have hydrogen bond contact with NAG 7 and a OH atom have hydrogen bond contact with C Chain of Pro 244 residue. Together, the study concludes that F127 has an excellent binding affinity towards the IgG complex system.

## Summary and outlook

Nanoparticles capable of *in vitro* and *in vivo* imaging could dissect cellular function at the molecular level and have been advancing as a potent diagnostic tool in precision medicine. While numerous reports focus on evaluating the staining capacity of quantum dot nanoparticles, their nano–bio interaction, which is critical for assessing their biocompatibility, is often overlooked. Quantum dot nanoparticles are the frontrunners as the diagnostic tools in studying the progression of inflammatory diseases like Rheumatoid arthritis, which are known to have elevated levels of immunoglobulins. Here, we produced water-borne, NIR-emitting SiQDs/F127 micelles capable of live cell imaging and studied their molecular interaction with human IgG through independent lines of evidence. The PLQY of the SiQDs/F127 micelles in water was 25% at pH 7. We first studied IgG's UV-visible absorption and fluorescence emission characteristics by gradually adding SiQDs/F127 micelles and *vice versa*. The binding parameters depended on IgG binding pockets/surface and the interaction between SiQDs/F127 micelles and IgG. Quenching constants at 10^10^th order was observed and resembled the creation of a rigid protein corona with strong association. The fluorescence spectral redshift (7 nm) further confirmed the strong protein corona formation. The CD spectroscopy results verified SiQD-De/F127 micelles as an excellent candidate to use in medical diagnosis with limited concentration levels without disturbing the IgG conformation. Accordingly, SiQD-De/F127 micelles demonstrated significant *in vitro* imaging capacity and limited cytotoxicity in the representative cell lines (HeLa and human dermal fibroblasts). Furthermore, the SiQD-De/F127 micelles displayed excellent *in vivo* imaging capacity even without requiring vectors and transfection agents when incubated with medaka fish eggs and larvae. It is important to note here that even at higher concentrations (1 μg mL^−1^), the normal embryonic development of medaka fish was not altered as the larvae hatched on day 17 from the micelles-treated egg as that of the control without any signs of toxicity. Therefore, SiQDs-De/F127 micelles are expected to have excellent biocompatibility for *in vivo* imaging studies. Our proof-of-concept study postulates the necessity to construct QDs without damaging the original protein conformations to preclude potential toxicity. In the future, this work will guide the development of new QDs as *in vitro* and *in vivo* diagnostic and therapeutic tools for inflammatory diseases such as rheumatoid arthritis.

## Conflicts of interest

The authors declare no competing financial interest.

## Supplementary Material

RA-013-D3RA00552F-s001

## References

[cit1] Chinnathambi S., Shirahata N. (2019). Recent advances on fluorescent biomarkers of near-infrared quantum dots for *in vitro* and *in vivo* imaging. Sci. Technol. Adv. Mater..

[cit2] Chandra S., Masuda Y., Shirahata N., Winnik F. M. (2017). Transition-metal-doped NIR-emitting silicon nanocrystals. Angew. Chem., Int. Ed..

[cit3] Chinnathambi S., Chen S., Ganesan S., Hanagata N. (2014). Silicon quantum dots for biological applications. Adv. Healthcare Mater..

[cit4] Tian M., Zhan J., Lin W. (2022). Single fluorescent probes enabling simultaneous visualization of duple organelles: Design principles, mechanisms, and applications. Coord. Chem. Rev..

[cit5] Chandra S., Ghosh B., Beaune G., Nagarajan U., Yasui T., Nakamura J., Tsuruoka T., Baba Y., Shirahata N., Winnik F. M. (2016). Functional double-shelled silicon nanocrystals for two-photon fluorescence cell imaging: spectral evolution and tuning. Nanoscale.

[cit6] Chinnathambi S., Hanagata N., Yamazaki T., Shirahata N. (2020). Nano–Bio Interaction between Blood Plasma Proteins and Water-Soluble Silicon Quantum Dots with Enabled Cellular Uptake and Minimal Cytotoxicity. Nanomaterials.

[cit7] Pandey S., Bodas D. (2020). High-quality quantum dots for multiplexed bioimaging: A critical review. Adv. Colloid Interface Sci..

[cit8] Chinnathambi S., Hanagata N. (2019). Photostability of quantum dot micelles under ultraviolet irradiation. Luminescence.

[cit9] Wagner A. M., Knipe J. M., Orive G., Peppas N. A. (2019). Quantum dots in biomedical applications. Acta Biomater..

[cit10] Zhang L., Wu Y., Luo X., Jia T., Li K., Zhou L., Mao Z., Huang P. (2022). A novel insight into mechanism of derangement of coagulation balance: interactions of quantum dots with coagulation-related proteins. Part. Fibre Toxicol..

[cit11] Liu J., Zhao W., Fan R.-L., Wang W.-H., Tian Z.-Q., Peng J., Pang D. W., Zhang Z. L. (2009). Investigation of the nonspecific interaction between quantum dots and immunoglobulin G using Rayleigh light scattering. Talanta.

[cit12] Gidwani B., Sahu V., Pandey S. S. S. R., Joshi V., Jain V. K., Vyas A. (2021). Quantum dots: Prospectives, toxicity, advances and applications. J. Drug Delivery Sci. Technol..

[cit13] Zhang H., Wang H., Yang H., Zhou D., Xia Q. (2021). Luminescent, protein-binding and imaging properties of hyper-stable water-soluble silicon quantum dots. J. Mol. Liq..

[cit14] Aho K., Heliovaara M., Knekt P., Reunanen A., Aromaa A., Leino A., Kurki P., Heikkila R., Palosuo T. (1997). Serum immunoglobulins and the risk of rheumatoid arthritis. Ann. Rheum. Dis..

[cit15] Schroeder Jr H. W., Cavacini L. (2010). Structure and Function of Immunoglobulins. J. Allergy Clin. Immunol..

[cit16] Zhang Y., Wu J. L. Y., Lazarovits J., Chan W. C. W. (2020). An Analysis of the Binding Function and Structural Organization of the Protein Corona. J. Am. Chem. Soc..

[cit17] Rampado R., Crotti S., Caliceti P., Pucciarelli S., Agostini M. (2020). Recent Advances in Understanding the Protein Corona of Nanoparticles and in the Formulation of “Stealthy” Nanomaterials. Front. Bioeng. Biotechnol..

[cit18] Rankl M., Ruckstuhl T., Artus G. R. J., Walser A., Seeger S. (2006). Conformational Reorientation of Immunoglobulin G During Nonspecific Interaction with Surfaces. ChemPhysChem.

[cit19] Yong K. T., Law W. C., Hu R., Ye L., Lie L., Swihart M. T., Prasad P. N. (2013). Nanotoxicity assessment of quantum dots: from cellular to primate studies. Chem. Soc. Rev..

[cit20] Lima T., Bernfur K., Vilanova M., Cedervall T. (2020). Understanding the Lipid and Protein Corona Formation on Different Sized Polymeric Nanoparticles. Sci. Rep..

[cit21] Chinnathambi S., Karthikeyan S., Hanagata N., Shirahata N. (2019). Molecular interaction of silicon quantum dot micelles with plasma proteins: hemoglobin and thrombin. RSC Adv..

[cit22] Chinnathambi S., Abu N., Hanagata N. (2017). CdSe/ZnS quantum dot micelles for long-term cell imaging without alteration to the native structure of the blood plasma protein human serum albumin. RSC Adv..

[cit23] Cukalevski R., Ferreira S. A., Dunning C. J., Berggård T., Cedervall T. (2015). IgG and fibrinogen driven nanoparticle aggregation. Nano Res..

[cit24] Hassan R. (2020). Interactions between Gold Nanoparticles and Immunoglobulin Isotypes. Electron. Theses Diss..

[cit25] Lin Z. Y., Ping T. U. L., Hui Z. Q., Gui K. X. (2013). Effect of protein molecules on the photoluminescence properties and stability of water-soluble CdSe/ZnS core-shell quantum dots. Chin. Sci. Bull..

[cit26] Naruse K. , Kinoshita M., Murata K. and Tanaka M., Medaka: Biology, management and experimental protocols, Wiley-Blackwell Publications, 2009, pp. 31–66, 10.1002/9780813818849

[cit27] Halgren T. A., Murphy R. B., Friesner R. A., Beard H. S., Frye L. L., Pollard W. T., Banks J. L. (2004). Glide: A New Approach for Rapid, Accurate Docking and Scoring. 2. Enrichment Factors in Database Screening. J. Med. Chem..

[cit28] Friesner R. A., Banks J. L., Murphy R. B., Halgren T. A., Klicic J. J., Mainz D. T., Repasky M. P., Knoll E. H., Shaw D. E., Shelley M., Perry J. K., Francis P., Shenkin P. S. (2004). Glide: A New Approach for Rapid, Accurate Docking and Scoring. 1. Method and Assessment of Docking Accuracy. J. Med. Chem..

[cit29] Farid R., Day T., Friesner R. A., Pearlstein R. A. (2006). New insights about HERG blockade obtained from protein modeling, potential energy mapping, and docking studies. Bioorg. Med. Chem..

[cit30] Sherman W., Day T., Jacobson M. P., Friesner R. A., Farid R. (2006). Novel Procedure for Modeling Ligand/Receptor Induced Fit Effects. J. Med. Chem..

[cit31] Sherman W., Beard H. S., Farid R. (2006). Use of an Induced Fit Receptor Structure in Virtual Screening. Chem. Biol. Drug Des..

[cit32] Biologics Suite 2018-2, Schrödinger, LLC, 2018, New York, NY, vol. 2

[cit33] Ghosh B., Hamaoka T., Nemoto Y., Takeguchia M., Shirahata N. (2018). Impact of anchoring monolayers on the enhancement of radiative recombination in light-emitting diodes based on silicon nanocrystals. J. Phys. Chem. C.

[cit34] Kortshagen U. R., Sankaran R. M., Pereira R. N., Girshick S. L., Wu J. J., Aydil E. S. (2016). Nonthermal plasma synthesis of nanocrystals: Fundamental principles, materials, and applications. Chem. Rev..

[cit35] Dohnalová K., Poddubny A.
N., Prokofiev A. A., de Boer W. D., Umesh C. P., Paulusse J. M. J., Zuilhof H., Gregorkiewicz T. (2013). Surface brightens up Si quantum dots: Direct bandgap-like size-tunable emission. Light: Sci. Appl..

[cit36] Zhang W., Shi Y., Chen Y., Ye J., Sha X., Fang X. (2011). Multifunctional Pluronic P123/F127 mixed polymeric micelles loaded with paclitaxel for the treatment of multidrug resistant tumors. Biomaterials.

[cit37] Paul B. K., Bhattacharjee K., Bose S., Guchhait N. (2012). A spectroscopic investigation on the interaction of a magnetic ferrofluid with a model plasma protein: Effect on the conformation and activity of the protein. Phys. Chem. Chem. Phys..

[cit38] Benesi H. A., Hildebrand J. H. (1949). A Spectrophotometric Investigation of the Interaction of Iodine with Aromatic Hydrocarbons. J. Am. Chem. Soc..

[cit39] Chinnathambi S., Velmurugan D., Hanagata N., Aruna P., Ganesan S. (2014). Investigations on the interactions of 5-fluorouracil with bovine serum albumin: Optical spectroscopic and molecular modeling studies. J. Lumin..

[cit40] Catherine F. D., Gemeinhart R. A. (2022). Biophysical Characterization of Interactions between Serum Albumin and Block Copolymer Micelles. ACS Biomater. Sci. Eng..

[cit41] Suryawanshi V. D., Anbhule P. V., Gore A. H., Patil S. R., Kolekar G. B. (2012). Spectroscopic Investigation on the Interaction of Pyrimidine Derivative, 2-Amino-6-hydroxy-4-(3,4-dimethoxyphenyl)-pyrimidine-5-carbonitrile with Human Serum Albumin: Mechanistic and Conformational Study. Ind. Eng. Chem. Res..

[cit42] Ware W. R. (1962). Oxygen quenching of fluorescence in solution-An experimental study of diffusion process. J. Phys. Chem..

[cit43] Karmakar S., Das T. K., Kundu S., Maiti S., Saha A. (2020). Physicochemical Understanding of Protein-Bound Quantum DotBased Sensitive Probing of Bilirubin: Validation with Real Samples and Implications of Protein Conformation in Sensing. ACS Appl. Bio Mater..

[cit44] Ghosh B., Takeguchi M., Nakamura J., Nemoto Y., Hamaoka T., Chandra S., Shirahata N. (2016). Origin of the photoluminescence quantum yields enhanced by alkane-termination of freestanding silicon nanocrystals: Temperature-dependence of optical properties. Sci. Rep..

[cit45] Micsonai A., Wien F., Kernya L., Lee Y.-H., Goto Y., Réfrégiers M., Kardos J. (2015). Accurate secondary structure prediction and fold recognition for circular dichroism spectroscopy. Proc. Natl. Acad. Sci. U. S. A..

[cit46] Micsonai A., Wien F., Bulyáki E., Kun J., Moussong E., Lee Y. H., Goto Y., Réfrégiers M., BeStSel J. K. (2018). A web server for accurate protein secondary structure prediction and fold recognition from the circular dichroism spectra. Nucleic Acids Res..

[cit47] Islam S., Moinuddin, Mir A. R., Raghav A., Habib S., Alam K., Ali. A. (2018). Glycation, Oxidation and Glycoxidation of IgG: A Biophysical, Biochemical, Immunological and Hematological study. J. Biomol. Struct. Dyn..

[cit48] Pramanik S., Hill S. K. E., Zhi B., Hudson-Smith N. V., Wu J. J., White J. N., McIntire E. A., Santosh V. S., Kondeti K., Lee A. L., Bruggeman P. J., Kortshagen U. R., Haynes C. L. (2018). Comparative toxicity assessment of novel Si quantum dots and their traditional Cd-based counterparts using bacteria models Shewanella oneidensis and Bacillus subtilis. Environ. Sci.: Nano.

[cit49] Sahay G., Batrakova E. V., Kabanov A. V. (2008). Different Internalization Pathways of Polymeric Micelles and Unimers and Their Effects on Vesicular Transport. Bioconjugate
Chem..

[cit50] Zastre J., Jackson J., Bajwa M., Liggins R., Iqbal F., Burt H. (2002). Enhanced cellular accumulation of a P-glycoprotein substrate, rhodamine-123, by Caco-2 cells using low molecular weight methoxypolyethylene glycol-block-polycaprolactone diblock copolymers. Eur. J. Pharm. Biopharm..

[cit51] Liang M., Lin I. C., Whittaker M. R., Minchin R. F., Monteiro M. J., Toth I. (2010). Cellular Uptake of Densely Packed Polymer Coatings on Gold Nanoparticles. ACS Nano.

[cit52] Phatvej W., Datta H. K., Wilkinson S. C., Mutch E., Daly A. K., Horrocks B. R. (2019). Endocytosis and Lack of Cytotoxicity of Alkyl-Capped Silicon Quantum Dots Prepared from Porous Silicon. Materials.

[cit53] Xiao Y., Forry S. P., Gao X., Holbrook R. D., Telford W. G., Tona A. (2010). Dynamics and mechanisms of quantum dot nanoparticle cellular uptake. J. Nanobiotechnol..

[cit54] Kwok K. W. H., Auffan M., Badireddy A. R., Nelson C. M., Wiesner M. R., Chilkoti A., Liu J., Marinakos S. M., Hinton D. E. (2012). Uptake of silver nanoparticles and toxicity to early life stages of Japanese medaka (Oryzias latipes): Effect of coating materials. Aquat. Toxicol..

[cit55] Kashiwada S. (2006). Distribution of Nanoparticles in the See-through Medaka (Oryzias latipes). Environ. Health Perspect..

[cit56] Yan N., Wang W. X. (2022). Maternal transfer and biodistribution of citrate and luminogens coated silver nanoparticles in medaka fish. J. Hazard. Mater..

