# Supplementary material for: Nano–bio interaction between human immunoglobulin G and nontoxic, near-infrared emitting water-borne silicon quantum dot micelles
Source: RSC Adv. 2023 Feb 20;13(9):6051–64. doi: 10.1039/d3ra00552f (PMC9939978; doi:10.1039/d3ra00552f)
Supplement: RA-013-D3RA00552F-s001 [file RA-013-D3RA00552F-s001.pdf]

Electronic Supplementary Material (ESI) for RSC Advances.
This journal is © The Royal Society of Chemistry 2023

Supporting Information

Nano-bio interaction between human immunoglobulin G and nontoxic, near-infrared emitting water-borne silicon quantum dot micelles

Shanmugavel Chinnathambi[1,2] *, Naoto Shirahata[3,4, 5, *], Mahima Kumar[1],

Subramani Karthikeyan[6], Katsuhiko Abe[1], Vaijayanthi Thangavel[1] and Ganesh N. Pandian[1*]

[1]Institute for Integrated Cell-Material Sciences (WPI-iCeMS), Institute for Advanced Study, Kyoto University, Kyoto 606-8501, Japan

[2]International Center for Young Scientists, National Institute for Materials Science (NIMS), 1-2-1 Sengen, Tsukuba, Ibaraki 305-0047, Japan

[3]Graduate School of Chemical Sciences and Engineering, Hokkaido University, Sapporo 060-0814, Japan

[4]International Center for Materials Nanoarchitectonics (WPI-MANA), NIMS, Namiki, Tsukuba 305-0044, Japan

[5]Department of Physics, Chuo University, 1-13-27 Kasuga, Bunkyo, Tokyo 112-8551, Japan

[6]Centre for Healthcare Advancement, Innovation and Research, Vellore Institute of Technology, Chennai, 600 127, India

C.A. E-mail: CHINNATHAMBI.Shanmugavel.8s@kyoto-u.ac.jp: SHIRAHATA.Naoto@ nims.go.jp; NAMASIVAYAM.ganeshpandian.5z@kyoto-u.ac.jp

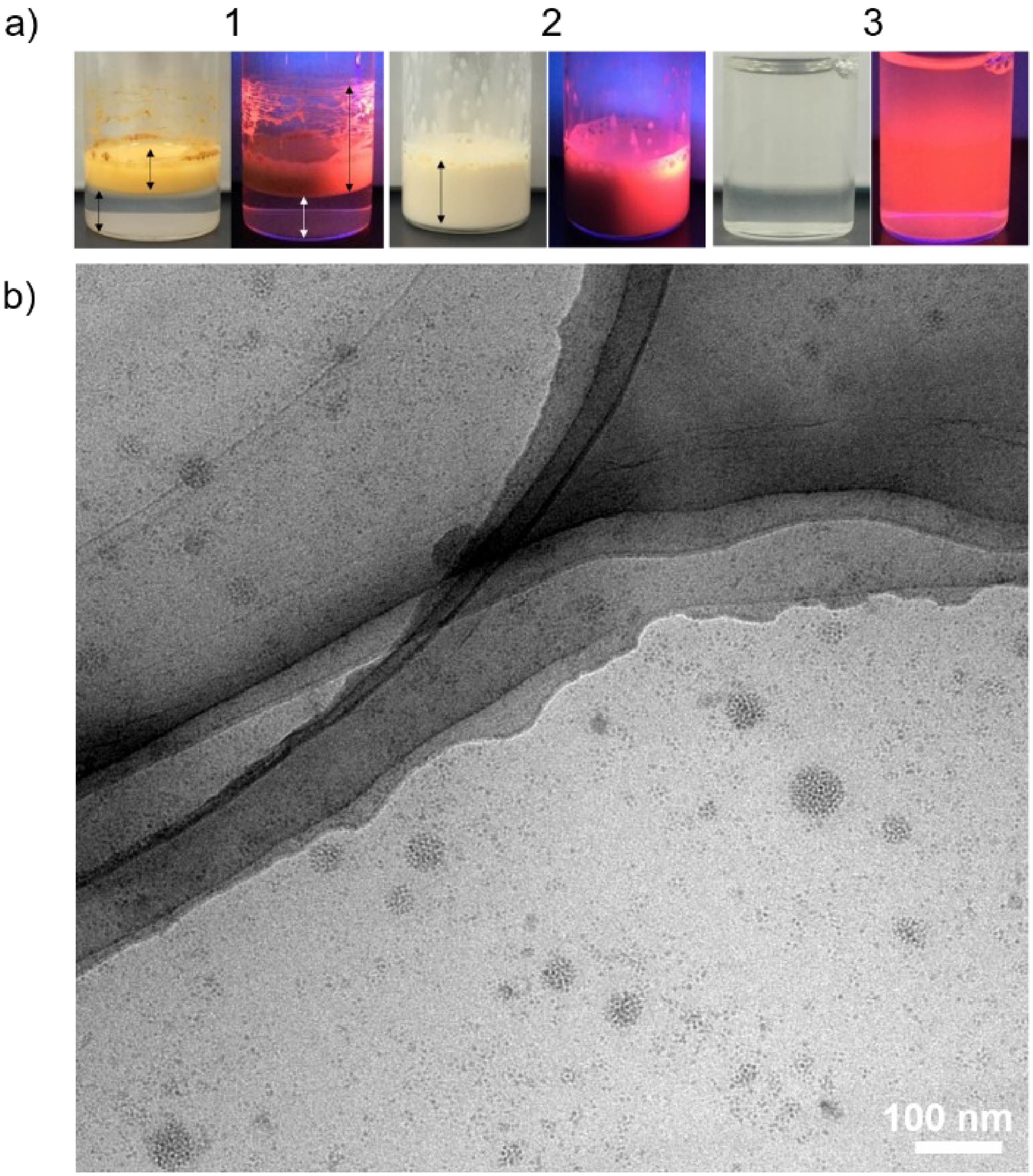

Figure S1. a). It is shown that SiQD-De/F127 nanoparticles can be prepared in three different ways. For each step, three sets of photos are shot using room lighting and UV light. Eight mL of Milli-Q water and 24 microliters of SiQD-De toluene were added to the glass bottle. Arrows on a left-pair image represent the amounts of toluene and water. b). An image of a SiQD-De/F127 nanoparticle TEM image. A Pluronic F127 molecule surrounds the constructed SiQDs-De cores, which range in diameter from 30 to 80 nm and are contrasted in the image.

The left-pair image was captured following toluene evaporation in the fume hood. A layer of SiQD-De (a yellow-colored band) has been left on top of the water, as is evident in the image on the left. SiQD-De is still present in small amounts inside the bottle's wall. The yellow-colored band in the right image of the pair's red-fluorescence confirms that it is made up of SiQD-De.

The middle-pair was obtained by shaking the glass bottle shown in the left-pair. The left image shows that the Si-De/F127 water solution is a milky color. The SiQD-De/F127 is well dispersed in the milky-colored solution, as seen in the right image.

The middle pair was diluted with Milli-Q water to produce the **right pair**. The left image shows a clear, colorless water solution. The successful encapsulation of SiQD-De with the Pluronic F127 molecule can be seen in the right image, which demonstrates that SiQD-De/F127 nanoparticles are highly dispersed in water.

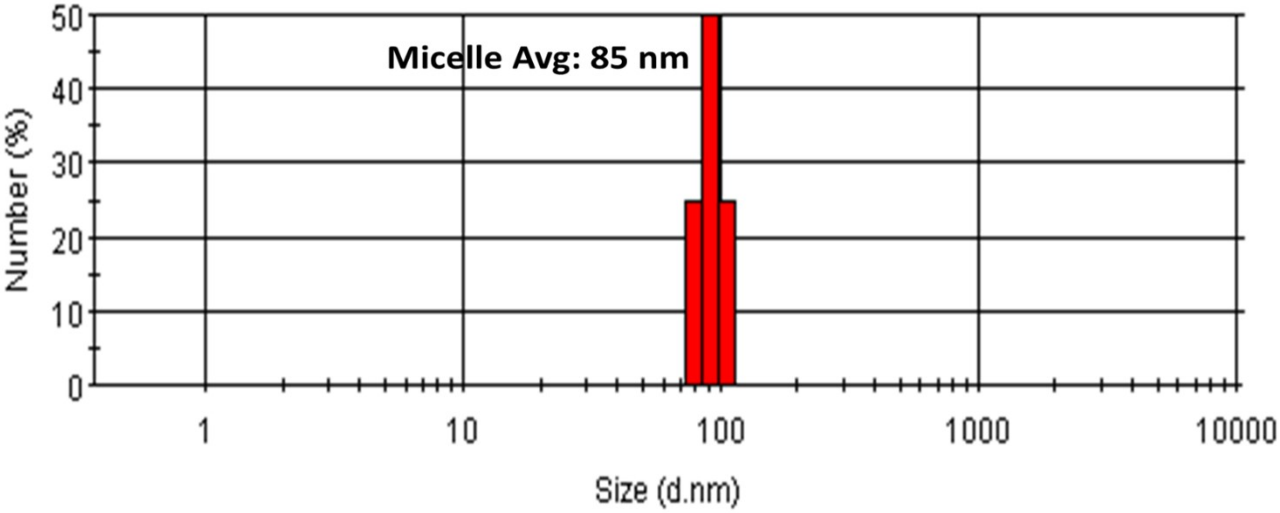

Figure S2. UV-visible absorption spectra, PL excitation (PLE) spectra, and PL spectra of two SiQD-De/F127 samples with different QD sizes.

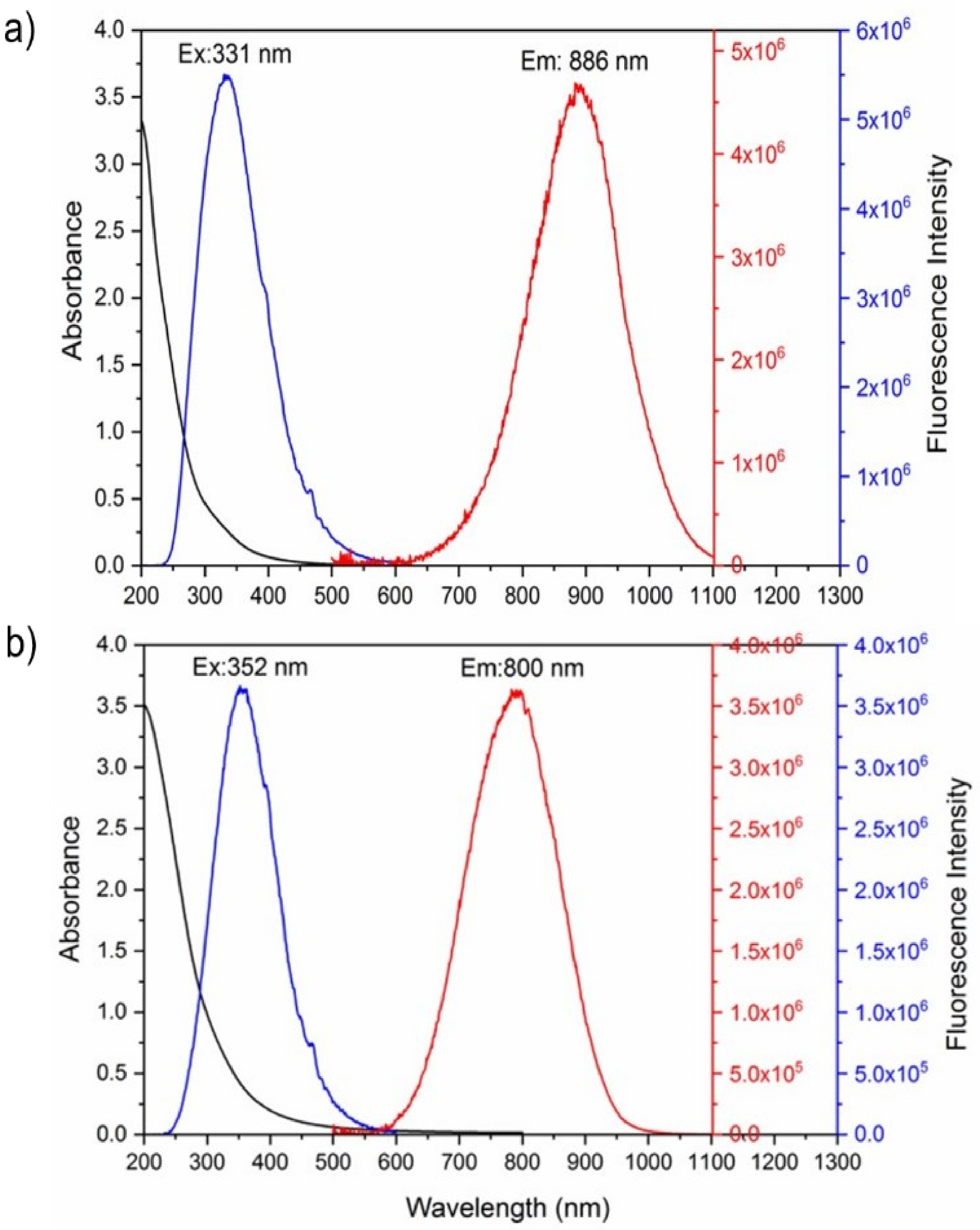

Figure S3. UV-visible absorption spectra, PL excitation (PLE) spectra, and PL spectra of two SiQD-De/F127 samples with different QD sizes. The diameters estimated from Scherrer broadening analysis are 4.8 nm, 4.0 nm. Both PLE spectra centered at 331, 352 nm, and their corresponding PL spectra centered at 886 and 800 nm spectrum are demonstrated for four SiQDs/F127 samples.

Table S1. The induced fit docking simulation produced 10 distinct binding poses. The gliding energy and docking score were used to determine the best positions.

	A	B	C	D	E	F	G	H	I	J	K	L	M	N	O
1	Stars	Title	Entry ID	Entry Nam	Date Added	Date Modified	PDB TITLE	PDB ID	PDB CLASS	PDB DEPO	PDB forma	PDB R	PDB Rfree	PDB RESOl	PDB EX
2		F127	1	F127.1	1/23/2023 10:57	1/23/2023 10:57	THE COMP	6KA7	IMMUNE S	21-Jun-19	3.3	0.257	0.333	3	X-RAY
3		F127	2	F127.1	1/23/2023 10:57	1/23/2023 10:57	THE COMP	6KA7	IMMUNE S	21-Jun-19	3.3	0.257	0.333	3	X-RAY
4		F127	3	F127.1	1/23/2023 10:57	1/23/2023 10:57	THE COMP	6KA7	IMMUNE S	21-Jun-19	3.3	0.257	0.333	3	X-RAY
5		F127	4	F127.1	1/23/2023 10:57	1/23/2023 10:57	THE COMP	6KA7	IMMUNE S	21-Jun-19	3.3	0.257	0.333	3	X-RAY
6		F127	5	F127.1	1/23/2023 10:57	1/23/2023 10:57	THE COMP	6KA7	IMMUNE S	21-Jun-19	3.3	0.257	0.333	3	X-RAY
7		F127	6	F127.1	1/23/2023 10:57	1/23/2023 10:57	THE COMP	6KA7	IMMUNE S	21-Jun-19	3.3	0.257	0.333	3	X-RAY
8		F127	7	F127.1	1/23/2023 10:57	1/23/2023 10:57	THE COMP	6KA7	IMMUNE S	21-Jun-19	3.3	0.257	0.333	3	X-RAY
9		F127	8	F127.1	1/23/2023 10:57	1/23/2023 10:57	THE COMP	6KA7	IMMUNE S	21-Jun-19	3.3	0.257	0.333	3	X-RAY
10		F127	9	F127.1	1/23/2023 10:57	1/23/2023 10:57	THE COMP	6KA7	IMMUNE S	21-Jun-19	3.3	0.257	0.333	3	X-RAY
11		F127	10	F127.1	1/23/2023 10:57	1/23/2023 10:57	THE COMP	6KA7	IMMUNE S	21-Jun-19	3.3	0.257	0.333	3	X-RAY
12															

	O	P	Q	R	S	T	U	V	W	X	Y	Z	AA	AB	AC	AD	AE	AF
1	PDB EXPDT	PDB EXPDT	PDB REMA	PDB REMA	Source Pat	Source File	Source File	entry id ba	prepared	assigned b	added hyd	treated me	created dis	deleted far	model num	Prime Coul	Prime Cova	Prime vdW
2	X-RAY DIFF	93	A, B, C, D,	1.000000 (	C:\Users\9	InducedFit	1	4	TRUE	TRUE	TRUE	TRUE	TRUE	TRUE	0	-26174.6	4212.04	-4270.51
3	X-RAY DIFF	93	A, B, C, D,	1.000000 (	C:\Users\9	InducedFit	2	4	TRUE	TRUE	TRUE	TRUE	TRUE	TRUE	0	-26167.2	4206.8	-4276.07
4	X-RAY DIFF	93	A, B, C, D,	1.000000 (	C:\Users\9	InducedFit	3	4	TRUE	TRUE	TRUE	TRUE	TRUE	TRUE	0	-26162.3	4207.25	-4269.24
5	X-RAY DIFF	93	A, B, C, D,	1.000000 (	C:\Users\9	InducedFit	4	4	TRUE	TRUE	TRUE	TRUE	TRUE	TRUE	0	-26154.7	4205.93	-4270.67
6	X-RAY DIFF	93	A, B, C, D,	1.000000 (	C:\Users\9	InducedFit	5	4	TRUE	TRUE	TRUE	TRUE	TRUE	TRUE	0	-26157.5	4206.19	-4274.9
7	X-RAY DIFF	93	A, B, C, D,	1.000000 (	C:\Users\9	InducedFit	6	4	TRUE	TRUE	TRUE	TRUE	TRUE	TRUE	0	-26162.5	4208.34	-4274.63
8	X-RAY DIFF	93	A, B, C, D,	1.000000 (	C:\Users\9	InducedFit	7	4	TRUE	TRUE	TRUE	TRUE	TRUE	TRUE	0	-26168.6	4213.64	-4278.5
9	X-RAY DIFF	93	A, B, C, D,	1.000000 (	C:\Users\9	InducedFit	8	4	TRUE	TRUE	TRUE	TRUE	TRUE	TRUE	0	-26152.4	4206.19	-4268.35
10	X-RAY DIFF	93	A, B, C, D,	1.000000 (	C:\Users\9	InducedFit	9	4	TRUE	TRUE	TRUE	TRUE	TRUE	TRUE	0	-26160.8	4211.15	-4272.08
11	X-RAY DIFF	93	A, B, C, D,	1.000000 (	C:\Users\9	InducedFit	10	4	TRUE	TRUE	TRUE	TRUE	TRUE	TRUE	0	-26161.4	4206.51	-4270.24

	AG	AH	AI	AJ	AK	AL	AM	AN	AO	AP	AQ	AR	AS	AT	AU	AV	AW	AX
1	Prime Solv	Prime Lipo	Prime Ener	Prime Hbo	Prime Pack	Prime Self(	ran protas	Potential E	RMS Deriv	Max Deriva	Stretch En	Bend Ener	LJ-14 Ener	El-14 Ener	Van der W	Electrostat	Dihedral E	ran impref
2	-4521.26	-4745.06	-36428.5	-304.493	-24.449	-600.213	TRUE	7.906	0	0	0.175	0.996	1.981	38.747	-1.188	-33.798	0.992	TRUE
3	-4530.07	-4743.68	-36438.6	-303.735	-24.449	-600.222	TRUE	7.906	0	0	0.175	0.996	1.981	38.747	-1.188	-33.798	0.992	TRUE
4	-4532.33	-4748.42	-36432.8	-303.029	-24.449	-600.337	TRUE	7.906	0	0	0.175	0.996	1.981	38.747	-1.188	-33.798	0.992	TRUE
5	-4536.86	-4746.45	-36430.3	-302.939	-24.449	-600.146	TRUE	7.906	0	0	0.175	0.996	1.981	38.747	-1.188	-33.798	0.992	TRUE
6	-4527.38	-4748	-36429.8	-303.499	-24.449	-600.265	TRUE	7.906	0	0	0.175	0.996	1.981	38.747	-1.188	-33.798	0.992	TRUE
7	-4525.2	-4745.54	-36427.4	-303.129	-24.449	-600.358	TRUE	7.906	0	0	0.175	0.996	1.981	38.747	-1.188	-33.798	0.992	TRUE
8	-4519.84	-4746.76	-36429.5	-304.662	-24.449	-600.293	TRUE	7.906	0	0	0.175	0.996	1.981	38.747	-1.188	-33.798	0.992	TRUE
9	-4534.44	-4743.32	-36420	-302.873	-24.449	-600.378	TRUE	7.906	0	0	0.175	0.996	1.981	38.747	-1.188	-33.798	0.992	TRUE
10	-4526.11	-4740.87	-36417.1	-303.618	-24.449	-600.323	TRUE	7.906	0	0	0.175	0.996	1.981	38.747	-1.188	-33.798	0.992	TRUE
11	-4522.95	-4742.84	-36420	-304.326	-24.449	-600.292	TRUE	7.906	0	0	0.175	0.996	1.981	38.747	-1.188	-33.798	0.992	TRUE

	AY	AZ	BA	BB	BC	BD	BE	BF	BG	BH	BI	BJ	BK	BL	BM	BN	BO	BP
1	grid versio	gridbox xc	gridbox yc	gridbox zc	gridbox xra	gridbox yra	gridbox zra	gridbox lig	gridbox lig	gridbox lig	receptor	glide gridfi	glide lignu	docking sc	glide ligand	glide ligand	glide ligand	glide gscor
2	66013	15.417	29.159	42.816	21.766	21.766	21.766	10	10	10	TRUE	InducedFit	1	1.513	0.138	0.306	0.445	1.513
3	66013	15.389	30.127	40.406	21.311	21.311	21.311	10	10	10	TRUE	InducedFit	1	2.071	0.188	0.419	0.61	2.071
4	66013	16.448	28.055	44.112	21.818	21.818	21.818	10	10	10	TRUE	InducedFit	1	1.884	0.171	0.381	0.554	1.884
5	66013	15.06	28.828	43.04	22.049	22.049	22.049	10	10	10	TRUE	InducedFit	1	1.759	0.16	0.356	0.518	1.759
6	66013	16.395	28.778	41.041	21.561	21.561	21.561	10	10	10	TRUE	InducedFit	1	1.879	0.171	0.38	0.553	1.879
7	66013	16.857	29.056	42.037	21.863	21.863	21.863	10	10	10	TRUE	InducedFit	1	2.145	0.195	0.434	0.631	2.145
8	66013	15.317	28.884	41.205	22.583	22.583	22.583	10	10	10	TRUE	InducedFit	1	2.525	0.23	0.51	0.743	2.525
9	66013	15.577	28.806	42.999	22.096	22.096	22.096	10	10	10	TRUE	InducedFit	1	2.204	0.2	0.446	0.649	2.204
10	66013	16.533	29.441	43.112	20.704	20.704	20.704	10	10	10	TRUE	InducedFit	1	2.457	0.223	0.497	0.723	2.457
11	66013	15.392	29.382	43.868	21.78	21.78	21.78	10	10	10	TRUE	InducedFit	1	2.659	0.242	0.538	0.783	2.659

BQ	BR	BS	BT	BU	BV	BW	BX	BY	BZ	CA	CB	CC	CD	CE	CF	CG	CH
glide lipo	glide hbon	glide meta	glide rewa	glide evdw	glide ecoul	glide erotb	glide esite	glide emoc	glide energ	glide einte	glide confr	glide poser	glide eff st	target tem	PDB CRYST	PDB CRYST	PDB CRYST
-0.699	-0.911	0	-2.25	-12.539	-12.713	7.907	0	-20.604	-25.252	4.02	181	278	0	298	59.926	107.42	171.35
-0.952	-0.29	0	-2.25	-14.226	-10.879	7.907	0	-18.808	-25.105	5.876	89	332	0	298	59.926	107.42	171.35
-1.33	-0.608	0	-2.25	-16.871	-6.608	7.907	0	-18.117	-23.479	5.309	68	310	0	298	59.926	107.42	171.35
-0.585	-0.585	0	-2.25	-8.438	-15.366	7.907	0	-19.064	-23.804	4.018	147	374	0	298	59.926	107.42	171.35
-0.806	-0.546	0	-2.25	-14.382	-11.371	7.907	0	-20.342	-25.753	4.897	51	283	0	298	59.926	107.42	171.35
-1.033	-0.575	0	-2.25	-15.091	-7.657	7.907	0	-18.817	-22.748	0.495	60	185	0	298	59.926	107.42	171.35
-0.858	0	0	-2.25	-15.895	-9.862	7.907	0	-18.203	-25.757	6.573	206	15	0	298	59.926	107.42	171.35
-1.045	-0.168	0	-2.25	-16.967	-9.27	7.907	0	-19.283	-26.237	6.971	109	145	0	298	59.926	107.42	171.35
-0.659	-0.553	0	-2.25	-13.967	-8.589	7.907	0	-16.691	-22.556	2.863	82	252	0	298	59.926	107.42	171.35
-0.332	-0.681	0	-2.25	-12.944	-8.912	7.907	0	-15.06	-21.856	4.697	105	171	0	298	59.926	107.42	171.35

CI	CJ	CK	CL	CM	CN
PDB CRYST	PDB CRYST	PDB CRYST	PDB CRYST	PDB CRYST	IFDScore
90	90	90	8	P 21 21 21	-1819.91
90	90	90	8	P 21 21 21	-1819.86
90	90	90	8	P 21 21 21	-1819.76
90	90	90	8	P 21 21 21	-1819.76
90	90	90	8	P 21 21 21	-1819.61
90	90	90	8	P 21 21 21	-1819.23
90	90	90	8	P 21 21 21	-1818.95
90	90	90	8	P 21 21 21	-1818.8
90	90	90	8	P 21 21 21	-1818.4
90	90	90	8	P 21 21 21	-1818.34
